# Supplementary material for: Advanced Vaginal Nanodelivery of Losartan Potassium via PEGylated Zein Nanoparticles for Methicillin-Resistant Staphylococcus aureus
Source: Pharmaceutics. 2025 Oct 18;17(10):1344. doi: 10.3390/pharmaceutics17101344 (PMC12567013; doi:10.3390/pharmaceutics17101344)
Supplement: Supplementary file 1 [file pharmaceutics-17-01344-s001.zip › pharmaceutics-3897646-supplementary.pdf]

Advanced Vaginal Nanodelivery of Losartan Potassium *via* PEGylated Zein Nanoparticles for Methicillin-Resistant *Staphylococcus aureus*

Rofida Albash [1,*], Mariam Hassan [2,3], Ahmed M. Agiba [4,*], Haneen Waleed Mohamed [5], Mohamed Safwat Hassan [5], Roaa Mohamed Ali [5], Yara E. Shalabi [5], Hend Mahmoud Abdelaziz Omran [5], Moaz A. Eltabeeb [6], Jawaher Abdullah Alamoudi [7], Asmaa Saleh [7], Amira B. Kassem [8], Yasmina Elmahboub [1]

[1] Department of Pharmaceutics, College of Pharmaceutical Sciences and Drug Manufacturing, Misr University for Science and Technology, Giza 12585, Egypt

[2] Department of Microbiology and Immunology, Faculty of Pharmacy, Cairo University, Cairo 11562, Egypt
[3] Department of Microbiology and Immunology, Faculty of Pharmacy, Galala University, Suez 43511, Egypt
[4] School of Engineering and Sciences, Tecnologico de Monterrey, Monterrey 64849, Mexico
[5] College of Pharmaceutical Sciences and Drug Manufacturing, Misr University for Science and Technology, Giza 12585, Egypt
[6] Department of Industrial Pharmacy, College of Pharmaceutical Sciences and Drug Manufacturing, Misr University for Science and Technology, Giza 12585, Egypt
[7] Department of Pharmaceutical Sciences, College of Pharmacy, Princess Nourah Bint Abdulrahman University, P.O. Box 84428, Riyadh 11671, Saudi Arabia
[8] Clinical Pharmacy and Pharmacy Practice Department, Faculty of Pharmacy, Damanhour University, Damanhour 22514, Egypt
* Correspondence: ahmed.agiba@tec.mx (A.M.A); rofida.albash@must.edu.eg (R.A)

Supplementary Information

Tables

Table S1. Chromatographic conditions for LOS quantification using HPLC.

**Parameter**	**Condition**
Instrument	Waters Alliance 2690 HPLC system, equipped with a 996-photodiode array (PDA) detector (Waters, Milford, MA, USA)
Column	Waters Xterra C_{18} (5 µm, 4.6 x 150 mm)
Mobile Phase	0.5% Triethylamine solution pH 2.4: Acetonitrile (60:40, v/v)
Flow Rate	1 mL/min
Detection Wavelength	225 nm
Injection Volume	20 µL
Column Temperature	20° C
Autosampler Temperature	25° C
Run Time	8.0 min

Table S2. Calibration Curve Parameters for LOS quantification using HPLC.

Conc (µg/mL)	Peak Area
0.02	2004
0.05	2740
0.1	7587
1	73260
10	755587
20	1537991
50	3841514
100	7728388

Figures

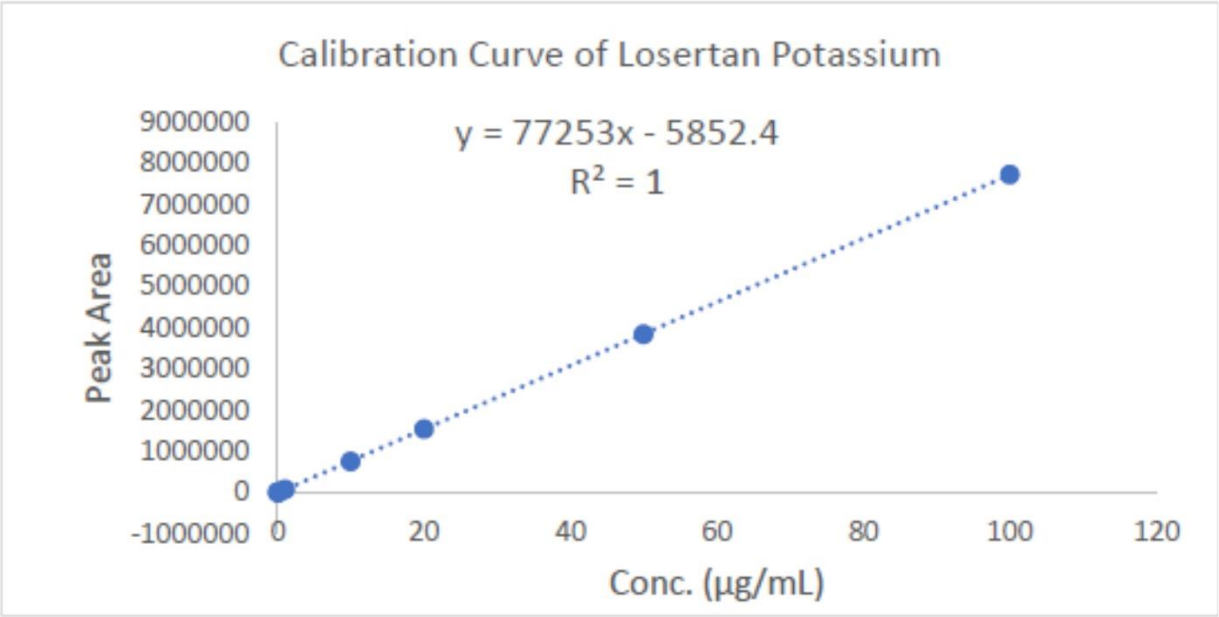

Figure S1. Calibration curve of LOS using HPLC within the concentration range of 0.02–100 µg/mL, showing excellent linearity ($R^2 = 1$).

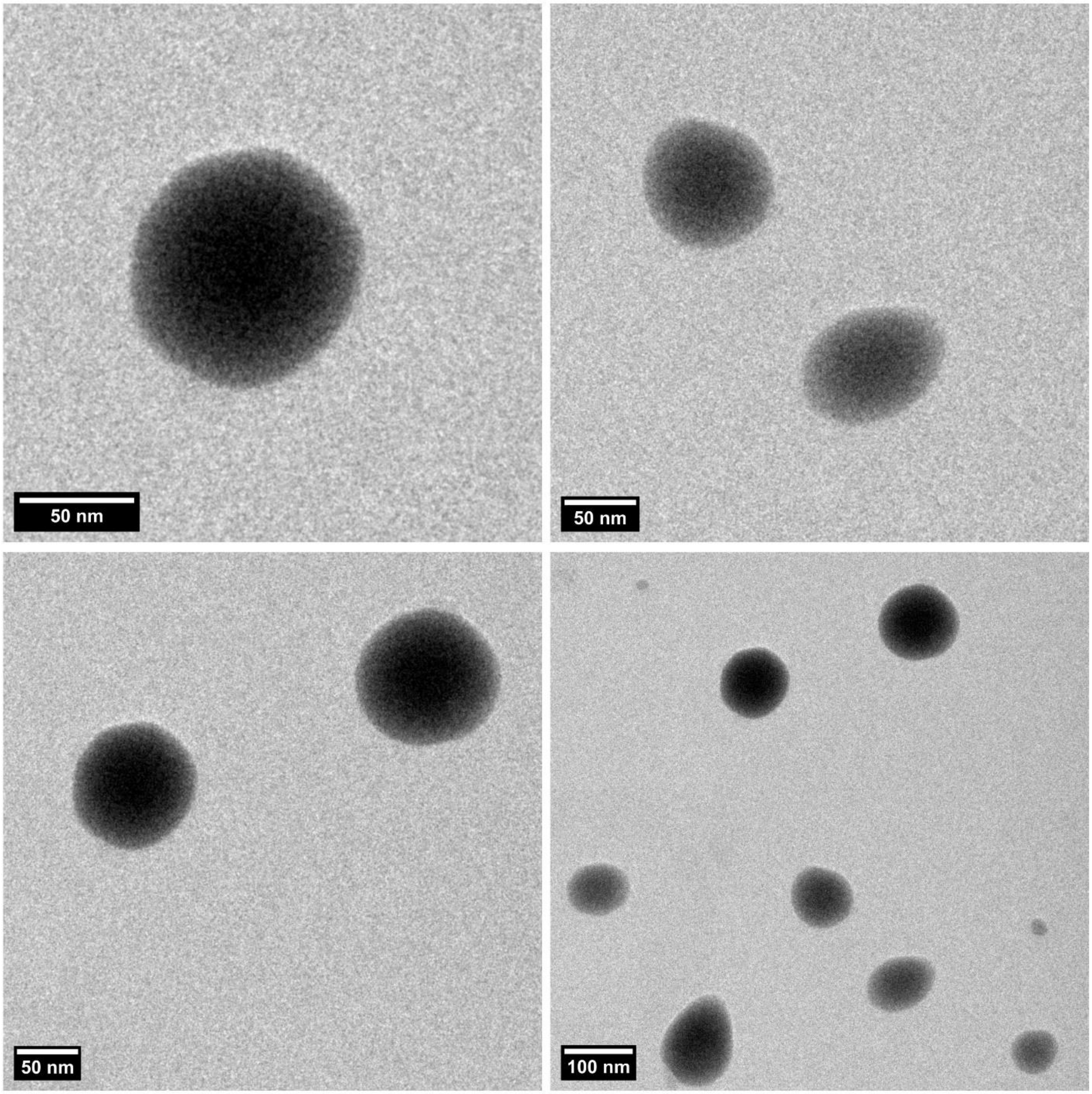

Figure S2. Transmission electron microscopy (TEM) images of the optimum formulation at magnifications corresponding to 50 nm and 100 nm scale bars.
